# Circuit quantum electrodynamics of granular aluminum resonators

**DOI:** 10.1038/s41467-018-06386-9

**Published:** 2018-09-24

**Authors:** N. Maleeva, L. Grünhaupt, T. Klein, F. Levy-Bertrand, O. Dupre, M. Calvo, F. Valenti, P. Winkel, F. Friedrich, W. Wernsdorfer, A. V. Ustinov, H. Rotzinger, A. Monfardini, M. V. Fistul, I. M. Pop

**Affiliations:** 1 Physikalisches Institut, Karlsruhe Institute of Technology, Wolfgang-Gaede-Str. 1, 76131 Karlsruhe, Germany; 2 Universite Grenoble Alpes, Institut NEEL, 25 rue des Martyrs BP 166, F-3800 Grenoble, France; 3 CNRS, Institut NEEL, 25 rue des Martyrs BP 166, F-3800 Grenoble, France; 4 Institute of Nanotechnology, Karlsruhe Institute of Technology, 76344 Eggenstein Leopoldshafen, Germany; 5 Russian Quantum Center, National University of Science and Technology MISIS, Leninskiy prsp., 4, 119049 Moscow, Russia; 6 Center for Theoretical Physics of Complex Systems, Institute for Basic Science, Expo-ro 55, Yuseong-gu, 34051 Daejeon Republic of Korea; 7 Institute of Nanotechnology, Karlsruhe Institute of Technology, Hermann-von-Helmholtz-Platz 1, 76344 Eggenstein Leopoldshafen, Germany

## Abstract

Granular aluminum (grAl) is a promising high kinetic inductance material for detectors, amplifiers, and qubits. Here we model the grAl structure, consisting of pure aluminum grains separated by thin aluminum oxide barriers, as a network of Josephson junctions, and we calculate the dispersion relation and nonlinearity (self-Kerr and cross-Kerr coefficients). To experimentally study the electrodynamics of grAl thin films, we measure microwave resonators with open-boundary conditions and test the theoretical predictions in two limits. For low frequencies, we use standard microwave reflection measurements in a low-loss environment. The measured low-frequency modes are in agreement with our dispersion relation model, and we observe self-Kerr coefficients within an order of magnitude from our calculation starting from the grAl microstructure. Using a high-frequency setup, we measure the plasma frequency of the film around 70 GHz, in agreement with the analytical prediction.

## Abstract (web summary)

The electrodynamics of superconducting devices make them suitable for applications as detectors, amplifiers, and qubits. Here the authors show that resonators made from granular aluminum, which naturally realizes a network of Josephson junctions, have practically useful impedances and nonlinearities.

## Introduction

The introduction of crystalline defects or dopants can give rise to so-called dirty superconductors^1^, characterized by reduced coherence length and quasiparticle mean free path. In particular, granular superconductors^2^ such as grAl^3,4^, consisting of remarkably uniform grains connected by Josephson contacts^5^ have attracted interest since the 60s, thanks to their rich phase diagram^6,7^ and practical advantages, like increased critical temperature^4,8^, critical field^9,10^, and kinetic inductance^11^. Here we report the measurement and modeling of circuit quantum electrodynamics^12^ properties of grAl microwave resonators in a wide frequency range, up to the spectral superconducting gap. Interestingly, we observe self-Kerr coefficients ranging from 10^−2^ Hz to 10^5^ Hz, within an order of magnitude from analytic calculations based on grAl microstructure. This amenable nonlinearity, combined with the relatively high-quality factors in the 10^5^ range, open new avenues for applications in quantum information processing^13^ and kinetic inductance detectors^14^.

Increasing the level of disorder in a superconducting material usually decreases the superfluid density and can induce a superconducting to insulating transition. Superconductors with low superconducting carrier density can exhibit rich physical properties, arising from a variety of phenomena such as quantum phase transitions^15^ and localization^2^. Granular aluminum is a typical example preferred by experimentalists, thanks to its relatively straightforward fabrication by aluminum evaporation in an oxygen atmosphere^3^, which can tune the film resistivity *ρ* over five orders of magnitude. The phase diagram of grAl thin films, with an initial increase of the critical temperature versus resistivity^16^, followed by a decrease and transition to an insulating state, has been extensively studied over the last 50 years, with notable recent developments in both theory^17^ and experiment^18,19^. These studies, mostly performed by direct current measurements, or broadband THz spectroscopy, offer a solid basis to start addressing the electrodynamics of grAl in the quantum regime, defined as the limit of single-photon excitations.

In the context of emerging quantum information platforms based on aluminum^13^, grAl provides precious ingredients such as low-loss and high-impedance environments, tolerance to high magnetic fields, or a robust source of nonlinearity. The prospect of implementing ultra-high impedance environments, at the level of the impedance quantum *R*_Q_ = *h*/(2*e*)^2^ ≃ 6.5 kΩ, for the design of qubits^20–22^ and parametric amplifiers^23^ or for the engineering of quantum states of light^24^ is very appealing. However, the electromagnetic properties of granular superconductors in the quantum regime are currently virtually unexplored.

Here we present a theoretical model and the corresponding experimental investigation of the dispersion relation and nonlinear Kerr coefficients for grAl resonators in the microwave regime. We will use the formalism of circuit quantum electrodynamics^12^ (cQED) and show that in a first-order approximation, the Hamiltonian of grAl, taking into account the interaction between the resonant modes, can be written in the familiar quantum optics form^25^

1 $$\begin{array}{*{20}{c}} {\frac{H}{\hbar } = \mathop {\sum}\limits_{n = 1} \left( {\omega _n + K_{nn}a_n^\dagger a_n} \right)a_n^\dagger a_n + \mathop {\sum}\limits_{\begin{array}{c}n,m = 1;n \ne m\end{array}} \frac{{K_{nm}}}{2}a_n^\dagger a_na_m^\dagger a_m.} \end{array}$$

The frequencies *ω*_*n*_ form the dispersion relation, the self-Kerr coefficients *K*_*nn*_ quantify the frequency shift of mode *n* for each added photon, and the cross-Kerr coefficients *K*_*nm*_, quantify the frequency shift of mode *n* for an added photon in mode *m*. The operators *a*_*n*_ and $a_n^\dagger$ are bosonic lowering and raising operators, and $a_n^\dagger a_n = N$ gives the photon number.

## Results

### Electrodynamic model

**Fig. 1:**
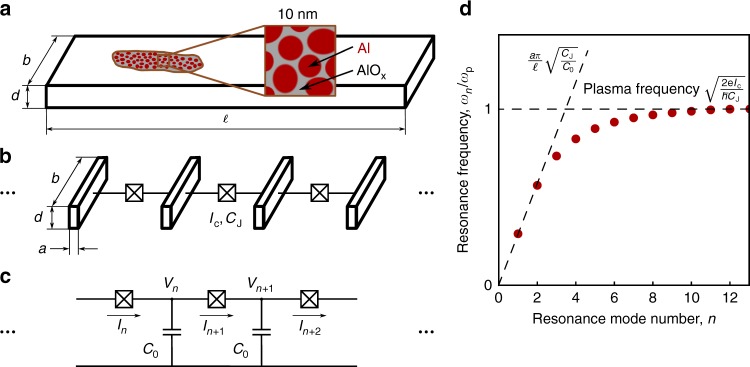
Schematic representation of a grAl stripline resonator with open-boundary conditions. **a** The length of the stripline, $\ell$, is in the range of mm, its width, *b*, is in the range of a few μm, and the thickness, *d*, is between 20 and 30 nm. Al grains (sketched in bordeaux color in the inset) have a diameter *a* = 3 ± 1 nm^4^. They are separated by aluminum oxide barriers (shown in gray), forming a 3D network of superconducting islands connected by Josephson contacts. **b** For the lowest-frequency standing-current modes along the stripline, the resonator can be modeled as a 1D array of effective Josephson junctions with critical current *I*_c_ and junction capacitance *C*_J_, corresponding to the summed critical currents and capacitances of the grains in a stripline section of length *a*. **c** The resulting circuit diagram consists of identical cells, each containing an effective JJ and the self capacitance *C*_0_ of the superconducting island. **d** Typical dispersion relation of a 1D JJ array, following Eq. (3). The spectrum saturates at the effective plasma frequency $\omega _{\mathrm{p}} = \sqrt {2eI_{\mathrm{c}}{\mathrm{/}}\hbar C_{\mathrm{J}}}$. The slope in the linear part of the dispersion relation is defined by the ratio ${\textstyle{{a\pi } \over \ell }}\sqrt {C_{\mathrm{J}}{\mathrm{/}}C_0}$

The microstructure of grAl consists of pure aluminum grains, with the average diameter *a*, separated by thin aluminum oxide barriers, as schematically illustrated in Fig. 1a. For films fabricated at room temperature with *ρ* > 10 μΩ cm, the grain size is homogeneous and independent of resistivity, *a* = 3 ± 1 nm^4^. We use grAl films with a resistivity between 40 μΩ cm and 4000 μΩ cm, below the superconducting to insulating transition at *ρ* ≃ 10^4^ μΩ cm^7^, and for which the kinetic inductance dominates over the geometric inductance^11^. We model this medium as a network of effective Josephson junctions (JJ), which provides a handle to calculate its dispersion relation^26^ and the Kerr coefficients^27,28^.

For elongated structures, such as stripline resonators (Fig. 1a), the calculation of the low-frequency dispersion relation and nonlinearity can be performed in the limit of one-dimensional (1D) current distributions along the stripline (see Supplementary Discussion), resulting in an effective JJ chain model (see Fig. 1b). The current is homogeneously distributed through the sample cross-section due to the fact that the thickness *d* ≃ 20 nm is much smaller than the magnetic field penetration depth, *λ*_L_ > 0.4 μm, depending on the film resistivity *ρ*, and the width *b* is smaller than the screening distance, $\lambda _ \bot = \lambda _{\mathrm{L}}^2{\mathrm{/}}d > 8$ μm^3^. The equivalent electrical schematics is shown in Fig. 1c, where each superconducting section of length *a* with self capacitance *C*_0_ is connected by effective JJs with critical current *I*_c_ and capacitance *C*_J_.

The classical equation of motion for the phase difference *φ*_*n*_ across the *n*th JJ is

2 $$\begin{array}{*{20}{c}} {2I_{\mathrm{c}}\,{\mathrm{sin}}\left( {\varphi _{n + 1}} \right) - I_{\mathrm{c}}\,{\mathrm{sin}}\left( {\varphi _{n + 2}} \right) - I_{\mathrm{c}}\,{\mathrm{sin}}\left( {\varphi _n} \right) + } \\ { + \frac{{\hbar C_{\mathrm{J}}}}{{2e}}\frac{{d^2}}{{dt^2}}\left( {2\varphi _{n + 1} - \varphi _{n + 2} - \varphi _n} \right) + } \\ { + \delta _{m,n}I_{{\mathrm{ext}}}\,{\mathrm{cos}}(\omega t) = \frac{{\hbar C_0}}{{2e}}\frac{{d^2\varphi _n}}{{dt^2}}.} \end{array}$$

The resonator drive is introduced as an external current applied to the *m*th cell, *δ*_*m*,*n*_*I*_ext_ cos(*ωt*), where *δ*_*m*,*n*_ is the Kronecker delta. In order to derive the eigenfrequencies, we use first-order Taylor expansion for the Josephson currents (see Supplementary Discussion). Thus we obtain the dispersion relation:

3 $$\omega _n = \frac{{na\pi }}{l}\sqrt {\frac{{2eI_{\mathrm{c}}}}{{\hbar \left( {C_0 + \frac{{n^2\pi ^2a^2}}{{l^2}}C_{\mathrm{J}}} \right)}}} ,$$

sketched in Fig. 1d, which is approximately linear for the lowest modes, and it saturates at the effective plasma frequency $\omega _{\mathrm{p}} = \omega _{n = \ell /a} = \sqrt {2eI_{\mathrm{c}}{\mathrm{/}}\hbar C_{\mathrm{J}}}$, as measured on mesoscopic JJ arrays^29^. As we will show in the following, the fundamental frequency *f*_1_ = *ω*_1_/2*π,* designed in the low GHz range, can provide a convenient link through the cross-Kerr effect to the higher modes of the dispersion relation, spanning up to ~100 GHz.

To derive the Kerr coefficients of the fundamental mode in Eq. (1), we solve the equation of motion, expanding the nonlinear terms up to third order. This method is similar to the one recently used to derive the nonlinearity of mesoscopic arrays of JJ^27,30^. By relating the phase response amplitude to the circulating photon number $\bar N$ (see Supplementary Discussion), we obtain the self-Kerr and cross-Kerr coefficients for the fundamental mode:

4 $$K_{1n} = {\cal C}\pi ea\frac{{\omega _1\omega _n}}{{j_{\mathrm{c}}V_{{\mathrm{grAl}}}}},\,{\mathrm{with}}\,n \ge 1.$$

Here, *e* is the electron charge, *a* is the grain size, *j*_c_ = *I*_c_/*bd* is the critical current density, *ω*_*n*_ are the eigenfrequencies given by Eq. (3), and $V_{{\mathrm{grAl}}} = bd\ell$ is the volume of grAl threaded by the current, see Fig. 1a. ${\cal C}$ is a numerical constant of order one, which for a sinusoidal current distribution is ${\cal C} = 3{\mathrm{/}}16$ for *n* = 1 and ${\cal C} = 1{\mathrm{/}}4$ for *n* > 1. Using the expression for the single-photon current as a function of frequency and total inductance, $I_{\bar N = 1}^2 = 2fh{\mathrm{/}}L$ and *L* ∝ 1/*j*_c_, Eq. (4), can be rewritten in a qualitatively similar form to the *K*_11_ coefficient estimated from Mattis–Bardeen theory for dirty superconductors^11,23^, $K_{11} \propto \left( {I_{\bar N = 1}{\mathrm{/}}I_ \ast } \right)^2$. The depairing current $I_ \ast$ is of the same order of magnitude as the critical current of the strip *I*_c_. In contrast, Eq. (4) offers a quantitative model for the nonlinearity of grAl, starting from the film properties. Remarkably, this analytic result agrees within an order of magnitude with the *K*_11_ coefficients measured on 14 grAl samples, spanning from *K*_11_ = 2 × 10^−2^ Hz to *K*_11_ = 3 × 10^4^ Hz.

Furthermore, the cross-Kerr coefficients, *K*_1*n*_, follow the functional dependence of the dispersion relation, *ω*_*n*_, given by Eq. (3) and reach a maximum at the effective plasma frequency *ω*_p_ (see Fig. 1d). Due to the high cross-Kerr interaction and high-mode density around *ω*_p_, we expect a strong response of the fundamental mode for drive frequencies in the vicinity of *ω*_p_/2*π*. As discussed in detail in the next section, for highly resistive samples (grAl#3) with *ρ* = 3000 μΩ cm, for which *ω*_p_ is low enough to be in the measurable range, we observe the expected plasma frequency response in the vicinity of 70 GHz.

### Measurements

**Fig. 2:**
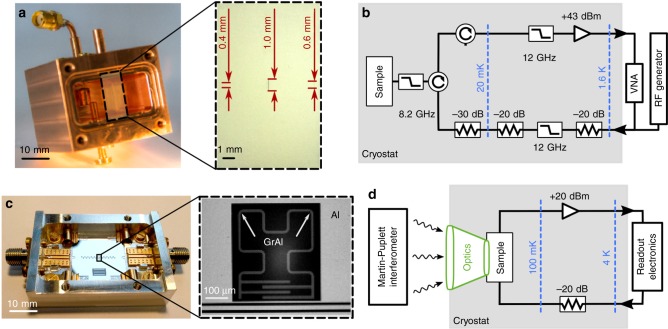
Two complementary microwave measurement techniques for the study of grAl resonators (grAl#1). Low-frequency setup: **a** Photograph of the Cu waveguide sample holder used to perform reflection measurements on stripline grAl resonators. The inset photograph shows three of the measured resonators, with dimensions 400 × 5.4 μm^2^, 600 × 10 μm^2^, and 1000 × 40 μm^2^. All resonators are 20-nm thick (see Supplementary Discussion). The waveguide is shielded and thermally anchored to the mixing chamber plate of a commercial dilution refrigerator. **b** Schematic of the cryogenic measurement setup. A reflection measurement with a vector network analyzer (VNA) characterizes the resonator response. The total attenuation on the input lines is −70 dB, and both input and output lines are interrupted by commercial and custom-made low-pass filters providing at least −30 dB of filtering above 9 GHz. The output signal is amplified by 40 dB, using a commercial high-electron mobility transistor amplifier. High-frequency setup: **c** Photograph of the Al sample holder and one of the resonators measured using a Martin–Puplett interferometer (MPI). The grAl resonators consist of a second-order Hilbert-shaped fractal inductor and an interdigitated capacitor. Twenty-two resonators are coupled to the common Al feed line, and each resonator is surrounded by an Al ground plane. Notice the different apparent color of the grAl film compared to Al. **d** Schematics of the measurement setup. The resonators are cooled down in a dilution refrigerator with optical access up to 200 GHz, facing the MPI^40^. The optics (shown in green) consist of a lens at room temperature, and two aperture and lens pairs at 4 K and 100 mK, in front of the sample^41^. The grAl resonator response to high-frequency illumination consists in shifting its low-frequency spectrum, which is continuously monitored in a transmission measurement through the common feed line. All samples were fabricated on c-plane, double side polished sapphire substrates, using standard e-beam and optical lithography lift-off techniques

To measure the dispersion relation, microwave losses, and the nonlinearity of grAl structures, we use three types of resonators of various shapes and sizes (see Methods), optimized for two complementary measurement setups (see Fig. 2), covering a broad frequency range up to 200 GHz.

**Fig. 3:**
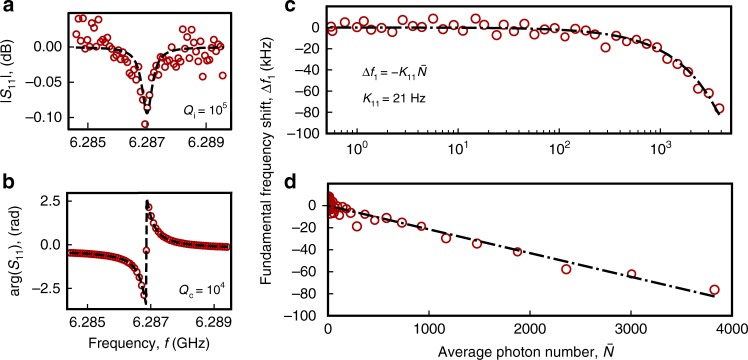
Measurement of the nonlinearity in grAl resonators. Typical measured amplitude (normalized by the sample holder response) (**a**) and phase (**b**) of the reflection coefficient *S*_11_ for resonator grAl#1 (see Fig. 2a). We typically observe internal quality factors of the resonators in the range of 10^5^. **c**, **d** We plot the measured shift of the first resonant frequency vs. circulating photon number $\bar N$ in logarithmic and linear scale, respectively. The corresponding self-Kerr coefficient extracted from the linear fit *K*_11_/2*π* = 21 Hz. The measured frequency shift remains linear versus photon number for all samples measured below bifurcation, which is consistent with the fact that the estimated circulating current never exceeds ~1% of the critical current

In Fig. 3a, b, we plot a typical amplitude and phase response measured for stripline resonators in the single-photon regime, $\bar N \approx 1$, which is relevant for quantum information applications. We extract an internal quality factor *Q*_i_ = 10^5^, comparable to values obtained for JJ array superinductances^29^. We obtain similar results for *Q*_i_ measurements on Hilbert-shaped (Fig. 2c) and aluminum-shunted stripline resonators, for tens of resonators, with grAl resistivities up to 4000 μΩ cm, corresponding to ~kΩ characteristic impedance. As discussed in ref.^31^, we estimate that *Q*_i_ is dominated by non-equilibrium quasiparticle dissipation, which could be suppressed by phonon and quasiparticle traps.

**Fig. 4:**
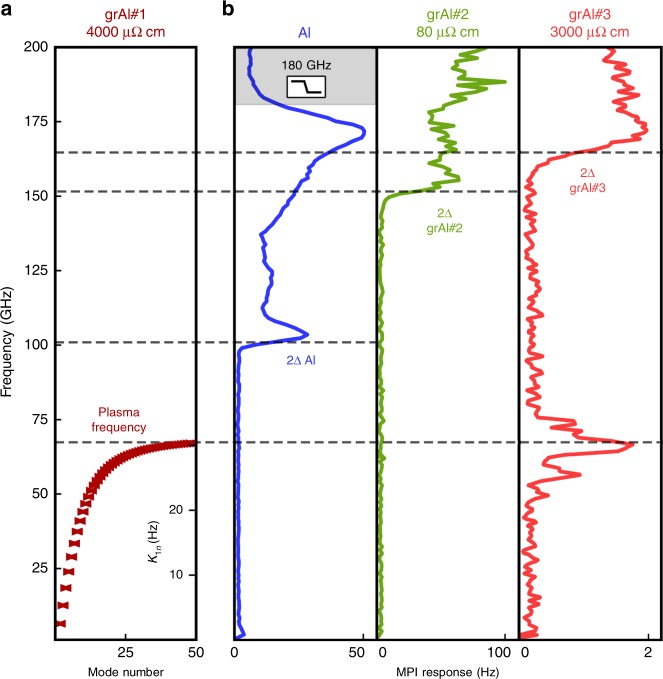
Measurement of the dispersion relation in grAl resonators. **a** Calculated dispersion relation *f*(*n*) for resonator grAl#1 starting from two-tone measurements of the third mode (see text). The spectrum saturates at the effective plasma frequency 68 ± 0.1 GHz. From Eq. (4), the cross-Kerr coefficients follow the dispersion relation, and their values are reported on the right axis. A significant cross-Kerr coupling enables the observation of the high-frequency spectrum up to the effective plasma frequency: photons populating the high end of the spectrum shift the low-lying eigen frequencies, which can be monitored via standard RF transmission measurements (see Fig. 2d). **b** Martin–Puplett Interferometer (MPI) response of Hilbert-shaped resonators made of 25-nm-thick Al, grAl with resistivity 80 μΩ cm (grAl#2), and grAl with resistivity 3000 μΩ cm (grAl#3). The illumination frequencies generated by the MPI range from a few GHz up to 200 GHz, with a resolution of 1 GHz. The different superconducting gaps of the films are evidenced by a strong MPI response due to quasiparticle excitation at 100 GHz for Al, at 150 GHz for grAl#2, and at 165 GHz for grAl#3. For the sample with the highest resistivity and the lowest critical current density, grAl#3, we observe a peak around 65 GHz, in the vicinity of the *ω*_p_ predicted from low-frequency measurements on sample grAl#1, with a similarly high resistivity (4000 μΩ cm, see text for details). This MPI response can be seen as the summed dispersive frequency shift due to cross-Kerr interactions *K*_1*n*_ between the fundamental mode and all higher populated modes

Using a two-tone spectroscopy, similar to a superconducting qubit readout procedure^12^, we measure higher modes of the dispersion relation for stripline resonators. Due to the symmetry of the electric field, the next mode, above the fundamental, coupled to the waveguide is the third. For sample grAl#1, we measure *f*_1_ = 6.287 GHz and *f*_3_ = 18.255 GHz. Notice that the dispersion relation already shows a measurable deviation from linear behavior, 3 × *f*_1_ − *f*_3_ = 606 ± 1 MHz, which, using Eq. (3), allows us to estimate an effective plasma frequency *ω*_p_ = 68 ± 0.1 GHz (see Supplementary Discussion), as shown in Fig. 4a).

Indeed, using a Martin–Puplett Interferometer (MPI) as a broadband illumination source up to 200 GHz and a Hilbert-shaped set of resonators (grAl#3) with similar sheet resistivity as grAl#1 mounted in an optical access cryostat, we observe a shift of the fundamental mode for illumination frequencies in the range 60–80 GHz (red curve in Fig. 4b). This shift is comparable to the pair-breaking response at twice the gap, and significantly above the noise floor. We interpret this response to be the cumulated cross-Kerr shift due to the population of the high-mode density region of the effective plasma frequency (see Fig. 4a).

As expected, for resonators with 50 times higher critical current densities *j*_c_, the effective plasma frequency can no longer be measured (green line in Fig. 4b), as it is above the spectroscopic gap frequency. To confirm the correct calibration of the MPI setup, we measured the response of a standard 25-nm aluminum film using an additional 180-GHz low-pass filter. The MPI measurements (blue line in Fig. 4b) indicate the expected Al spectral gap value of 100 GHz, above which the illumination can break the Cooper pairs, inducing a shift of the fundamental mode and a *Q*_i_ decrease^14^. Finally, notice that the spectroscopic gap of samples grAl#2 and grAl#3 increases with resistivity, as expected^7^.

**Fig. 5:**
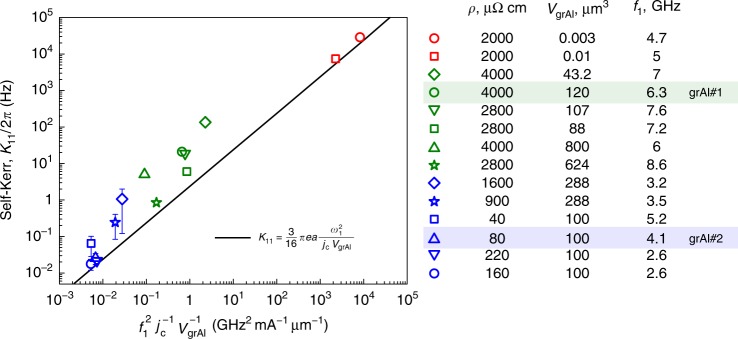
Measured grAl self-Kerr nonlinearity. The measured self-Kerr coefficients of 14 grAl samples are plotted versus $f_1^2{\mathrm{/}}j_{\mathrm{c}}V_{{\mathrm{grAl}}}$, where *f*_1_ = *ω*_1_/2*π* is the frequency of the first-mode, *j*_c_ is the critical current density, and *V*_grAl_ is the sample volume, with values listed for each sample in the legend. Hilbert-shaped resonator samples are represented in blue, stripline samples in green, and Al-shunted stripline resonators in red. The error bars for the blue points show the standard deviation of the measured *K*_11_ for nominally identical resonators. In the legend, the samples are listed in decreasing *K*_11_ order, within each group. The blue up-oriented triangle corresponds to sample grAl#2 (highlighted in blue), and the green circle corresponds to grAl#1 (highlighted in green). The black line shows the calculated self-Kerr from Eq. (4), for a grain size *a* = 4 nm, which includes the 1 nm thickness of the aluminum oxide barrier. We estimate the main error source to be the photon number calibration, which can only be estimated within a factor of 10

To measure the self-Kerr coefficient, *K*_11_, we monitor the fundamental frequency as a function of photon population $\bar N$ using the low-frequency setup (Fig. 2b). Typical measurement results are shown in Fig. 3c, d in linear and logarithmic scale, respectively. In Fig. 5, we report the measured *K*_11_ for 14 types of grAl resonators, grouped in three different geometries: KID (in blue), striplines (in green), and Al-shunted striplines (in red); details on the resonators' geometry are given in the Supplementary Discussion. For a direct comparison with Eq. (4) represented by the black line, we plot the measured self-Kerr coefficients versus$f_1^2{\mathrm{/}}j_{\mathrm{c}}V_{{\mathrm{grAl}}}$ using a measured *j*_c_ = 1.1 mA/μm^2^ for *ρ* = 1600 μΩ cm (see Supplementary Discussion) and scaling it according to *j*_c_ ∝ 1/*ρ* for all resistivities^7^. We would like to emphasize that there are no fitting parameters. We estimate the main source of error, responsible for the scatter of the points and for the deviation compared to Eq. (4) to be the photon number calibration. We can only perform this calibration by estimating the total attenuation of the input lines at various frequencies. We estimate this method to be accurate only within a factor of 10. Remarkably, the self-Kerr coefficient of grAl can be tuned over six orders of magnitude by varying the room temperature resistivity *ρ* ∝ 1/*j*_c_ and the resonator volume *V*_grAl_, without compromising the internal quality factor.

## Discussion

According to the strength of the nonlinearity, we can divide the possible grAl applications into three categories. First, for superinductors^29,32–34^ and microwave kinetic inductance detectors^14,35,36^, the nonlinearity should be as low as possible. The devices plotted in green in Fig. 5 could be used as superinductors with a self-Kerr coefficient of only tens of Hz, which would be at least three orders of magnitude lower than the state-of-the-art^27^. Second, for parametric devices, such as amplifiers^23^ or frequency converters^37,38^, the self-Kerr coefficient should be in the kHz range, as shown by the devices plotted in red in Fig. 5. Fabricating them using grAl instead of mesoscopic JJ arrays offers the advantages of compactness and single-step fabrication. Finally, in the case of transmon qubits^39^, the self-Kerr nonlinearity should be even higher, in the tens of MHz range, which could be achieved by reducing the grAl volume and increasing the resistivity of the film.

In conclusion, granular aluminum is a superconductor with high characteristic impedance, low microwave losses, and amenable nonlinearity, which recommend it as a material of choice for quantum information processing. Using a high-frequency setup, including a Martin–Pupplet interferometer, we observe the effective plasma frequency of highly inductive grAl devices in the range of 70 GHz, which is in agreement with estimates based on a 1D JJ array model and the measured low-frequency spectrum. The measured self-Kerr coefficients agree within an order of magnitude with our analytic model, and they are in the range of applications for parametrically pumped devices, such as quantum amplifiers. Highly inductive grAl films could implement low-loss superinductors for quantum circuits or ultra-sensitive kinetic inductance detectors.

## Methods

### Experimental apparatus

The dispersion relation for grAl resonators spans up to ~100 GHz. To cover this wide frequency range, we employ two complementary measurement setups, and we use the first mode as a link between them, via the cross-Kerr effect.

### Low-frequency setup

The low-frequency part of the spectrum (*n* = 1–3), up to 20 GHz, is measured using microwave transmission and reflection measurements in a standard cQED setup^12^ (Fig. 2b). The grAl stripline resonators (Fig. 2a) are mounted in a 3D waveguide (WG) sample holder, housed inside a hermetic copper shield coated with infrared-absorbing material. In this low-noise setup, all microwave lines are filtered above 8 GHz using commercial low-pass filters, circulators, and infrared absorbers identical to the setup in ref. ^31^ in order to reduce stray radiation. Even though the Hilbert-shaped grAl resonators and their aluminum sample holder (Fig. 2c) are designed to operate as kinetic inductance detectors (KIDs), which are required for the measurement of their high-frequency spectrum by means of direct optical spectroscopy (Fig. 2d), they were also measured by standard microwave transmission in the low-noise, shielded setup of Fig. 2b. The high level of filtering and superior shielding, offered by the measurement setup optimized for low frequencies, is required for the protection of the fundamental mode against stray excitations, which is essential for the measurement of its coherence and nonlinear properties (self-Kerr and cross-Kerr).

### High-frequency setup

For the measurement of the effective plasma frequency, we use the wide-frequency band setup of Fig. 2d, consisting of an optical access cryostat coupled to a Martin–Puplett Interferometer (see Supplementary Discussion). The fundamental mode is continuously measured via microwave transmission measurements, while its frequency is shifted by cross-Kerr interactions with optically populated higher modes of the dispersion relation.

## Electronic supplementary material

Supplementary Information

## Data Availability

All relevant data are available from the authors.
