# Supplementary material for: Circuit quantum electrodynamics of granular aluminum resonators
Source: Nat Commun. 2018 Sep 24;9:3889. doi: 10.1038/s41467-018-06386-9 (PMC6155321; doi:10.1038/s41467-018-06386-9)
Supplement: Supplementary file 1 — Supplementary Information [file 41467_2018_6386_MOESM1_ESM.pdf]

Supplementary Information

Circuit Quantum Electrodynamics of Granular Aluminum Resonators

Maleeva et al.

SUPPLEMENTARY DISCUSSION

Current distribution

We perform all calculations in the limit of a one dimensional current distribution along the resonator. Here we present the results of a finite elements simulation of the current distribution for the five lowest stripline resonator modes. Supplementary Figure 1 shows the current distribution along (top row) and across (bottom row) the stripline resonator. The current along the resonator is at least two orders of magnitude higher than the current in the perpendicular direction. This validates the 1D current distribution assumption and allows us to exclude drum-like modes as a source of deviation from the linear dispersion relation.

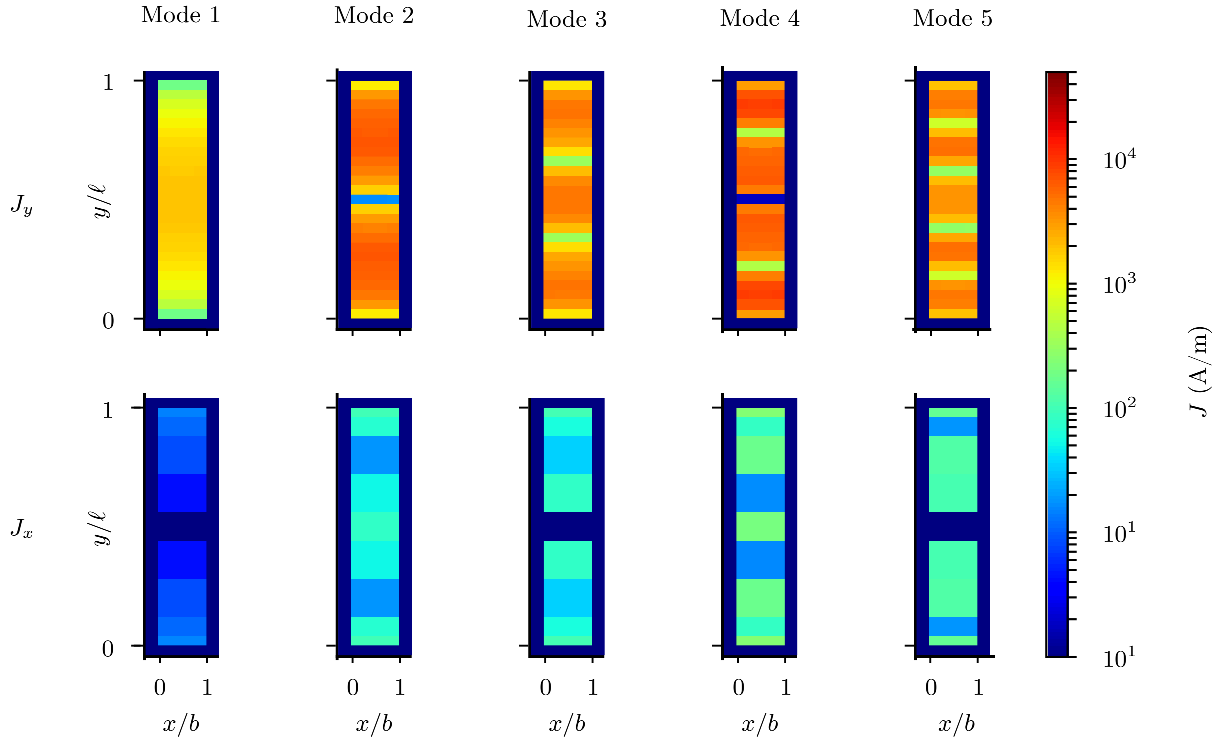

Supplementary Figure 1. **Current distribution.** Finite elements method simulation of surface current density in a high kinetic inductance $\lambda/2$ stripline resonator with dimensions 40×1000 µm^2. The top row shows the current density J_y along the y-dimension ($\ell = 1000$ µm) for the first five modes. The corresponding current density J_x along the x-dimension ($b = 40$ µm) is shown in the bottom row. J_y is at least two orders of magnitude larger than J_x for all modes. Therefore, we conclude that up to the fifth mode the resonator follows the well known $\lambda/2$ current distribution, and that the measured nonlinear dispersion relation of the third mode, reported in the main text, is not caused by a drum mode like behavior.

Details on analytical model

In order to derive the equation of motion for a 1D JJ array we write the Kirchhoff laws and Josephson equations for two neighboring effective junctions as

$$
\begin{aligned}
I_n &= I_{n+1} + C_0 \frac{dV_n}{dt}, \\
I_{n+1} &= I_{n+2} + C_0 \frac{dV_{n+1}}{dt}, \\
V_{n+1} - V_n &= \frac{\hbar}{2e} \frac{d(\chi_{n+1} - \chi_n)}{dt},
\end{aligned} \tag{1}
$$

where V_n and χ_n are the voltage and the phase on the nth node respectively, and I_n is the current through the nth JJ. Combining these equations and introducing an excitation as an external current $I_{\text{ext}} \cos(\omega t)$ applied to the mth cell, where $x_m = l/2$, we obtain

$$
2I_{n+1} - I_{n+2} - I_n + \delta_{m,n} I_{\text{ext}} \cos(\omega t) = \frac{\hbar C_0}{2e} \frac{d^2 \varphi_n}{dt^2}, \tag{2}
$$

where $\varphi_n = \chi_{n+1} - \chi_n$ is the phase difference across the nth JJ. The JJ current is described as the following

$$
I_n = I_{\text{c}} \sin(\varphi_n) + \frac{\hbar C_{\text{J}}}{2e} \frac{d^2(\varphi_n)}{dt^2}. \tag{3}
$$

Substituting this expression to Supplementary Eq. (2) we obtain the equation of motion in the discrete limit

$$
\begin{aligned}
& 2I_{\text{c}} \sin(\varphi_{n+1}) - I_{\text{c}} \sin(\varphi_{n+2}) - I_{\text{c}} \sin(\varphi_n) + \\
& + \frac{\hbar C_{\text{J}}}{2e} \frac{d^2}{dt^2} (2\varphi_{n+1} - \varphi_{n+2} - \varphi_n) + \\
& + \delta_{m,n} I_{\text{ext}} \cos(\omega t) = \frac{\hbar C_0}{2e} \frac{d^2 \varphi_n}{dt^2}.
\end{aligned} \tag{4}
$$

In order to obtain the dispersion relation of the resonator we rewrite Supplementary Eq. (4) in the continuous limit

$$
\begin{aligned}
& I_{\text{c}} a^2 \frac{d^2}{dx^2} \sin \varphi(x,t) + \frac{\hbar C_{\text{J}}}{2e} a^2 \frac{d^2}{dt^2} \frac{d^2}{dx^2} \varphi(x,t) + \\
& + a\delta\left(x - \frac{\ell}{2}\right) I_{\text{ext}} \cos(\omega t) = \frac{\hbar C_0}{2e} \frac{d^2}{dt^2} \varphi(x,t).
\end{aligned} \tag{5}
$$

We consider here the first resonance mode with sinusoidal current distribution $I(x,t) = I(t) \sin\left(\frac{\pi x}{\ell}\right)$; the corresponding phase difference is $\varphi(x,t) = \varphi(t) \sin \frac{\pi x}{\ell}$. By substituting the phase difference ansatz in Supplementary Eq. (5), multiplying the equation by $\sin \frac{\pi x}{\ell}$ and integrating it along the resonator, we obtain the equation of motion of the resonator

$$
\frac{\hbar}{2e} \left(C_0 + \frac{\pi^2 a^2}{\ell^2} C_{\text{J}}\right) \frac{d^2 \varphi(t)}{dt^2} + 2I_{\text{c}} \frac{\pi^2 a^2}{\ell^2} J_1[\varphi(t)] = \frac{2a}{\ell} I_{\text{ext}} \cos(\omega t). \tag{6}
$$

We approximate the Bessel function to first order $J_1[\varphi(t)] \sim \varphi(t)/2$, thus obtaining

$$
\alpha I_{\text{c}} \frac{\pi^2 a^2}{\ell^2} \varphi(t) + \frac{\hbar}{2e} \left(C_0 + \frac{\pi^2 a^2}{\ell^2} C_{\text{J}}\right) \frac{d^2 \varphi(t)}{dt^2} = \frac{2a}{\ell} I_{\text{ext}} \cos(\omega t). \tag{7}
$$

in the linear limit. Notably this equation is very similar to the motion equation of a current biased JJ. Solving Supplementary Eq. (7) we obtain the first resonance frequency of our system

$$
\omega_1 = \frac{a\pi}{l} \sqrt{\frac{2eI_{\text{c}}}{\hbar \left(C_0 + \frac{\pi^2 a^2}{l^2} C_{\text{J}}\right)}}. \tag{8}
$$

Performing the same calculations using the coordinate distribution of the higher resonance modes, we obtain the dispersion relation

$$\omega_n = \frac{na\pi}{l}\sqrt{\frac{2eI_{\mathrm{c}}}{\hbar\left(C_0 + \frac{n^2\pi^2a^2}{l^2}C_{\mathrm{J}}\right)}}. \tag{9}$$

In order to derive the self-Kerr coefficient of the fundamental mode we solve the nonlinear equation of motion. The coupling of our resonator to the environment and internal losses of the resonator are introduced to Supplementary Eq. (6) as a damping term with a parameter $\gamma = \omega_1/Q_{total}$ ($\sim 10^4 - 10^5$ according to the experiment)

$$\ddot{\varphi}(t) + \frac{4e\tilde{I}_{\mathrm{c}}}{\hbar\tilde{C}}J_1[\varphi(t)] + \gamma\dot{\varphi}(t) = \frac{4e}{\hbar\tilde{C}}\frac{a}{\ell}I_{\mathrm{ext}}\cos(\omega t), \tag{10}$$

where $\tilde{I}_{\mathrm{c}} = I_{\mathrm{c}}\frac{\pi^2a^2}{\ell^2}$ and $\tilde{C} = C_0 + \frac{\pi^2a^2}{\ell^2}C_{\mathrm{J}}$. Since we are working in resonance regime, we require the system to oscillate only with the driving frequency ω by assuming

$$\varphi = \varphi_a\cos(\omega t + \delta), \tag{11}$$

where φ_a is the amplitude of the response for each JJ and δ is a phase delay due to losses in the system. Solving Supplementary Eq. (10) with ansatz (11) we obtain

$$\varphi_a = \frac{\frac{4e}{\hbar\tilde{C}}\frac{a}{\ell}I}{\sqrt{\left(\omega^2 - 4\omega_1^2J_0[\varphi_a/2]J_1[\varphi_a/2]/\varphi_a\right)^2 + \gamma^2\omega^2}}. \tag{12}$$

For small φ_a we can expand the Bessel functions in series up to the third order, thus obtaining

$$\varphi_a = \frac{\frac{4e}{\hbar\tilde{C}}\frac{a}{\ell}I_{\mathrm{ext}}}{\sqrt{\left(\omega^2 - \omega_1^2\left(1 - \frac{3\varphi_a^2}{32}\right)\right)^2 + \gamma^2\omega^2}}. \tag{13}$$

Since at resonance the response φ_a reaches its highest value, we derive the resonance frequency of the nonlinear resonator by maximizing Supplementary Eq. (13).

$$\omega = \omega_1\sqrt{1 - \frac{3\varphi_a^2}{32}} \simeq \omega_1\left(1 - \frac{3\varphi_a^2}{64}\right). \tag{14}$$

One can see that in comparison to a single JJ with the resonance frequency, $\omega = \omega_1\left(1 - \frac{\varphi_a^2}{4}\right)$, the 1D array has similar, but lower first order nonlinearity. By relating the phase response to an average circulating photon number $\bar{N}$ (see Appendix), we obtain the self-Kerr coefficient for the fundamental mode

$$K_{11} = \frac{3}{16}\pi ea\frac{\omega_1^2}{j_cV_{\mathrm{grAl}}}. \tag{15}$$

In the following, we consider the cross-Kerr coupling between two different modes m and k with eigenfrequencies ω_a and ω_b. In order to obtain the cross-Kerr coefficients K_{mk} one needs to solve the equation of motion with excitation terms $a\delta\left(x - \frac{\ell}{2}\right)(I_m\cos(\omega_at) + I_k\cos(\omega_bt))$, representing the drive of mth and k^{th} modes

$$\begin{aligned} I_{\mathrm{c}}a^2\frac{d^2}{dx^2}\sin\varphi(x,t) + \frac{\hbar C_{\mathrm{J}}}{2e}a^2\frac{d^2}{dt^2}\frac{d^2}{dx^2}\varphi(x,t) + \\ + a\delta\left(x - \frac{\ell}{2}\right)(I_m\cos(\omega_at) + I_k\cos(\omega_bt)) = \frac{\hbar C_0}{2e}\frac{d^2}{dt^2}\varphi(x,t). \end{aligned} \tag{16}$$

We are mainly interested in the cross-Kerr coupling of the fundamental mode and we start with considering the coupling between the first and third modes. Similarly to the self-Kerr case, we look for a solution of the equation as a sum of the two driven modes

$$\varphi(x,t) = \varphi_1(t)\sin\frac{\pi x}{\ell} + \varphi_3(t)\sin\frac{3\pi x}{\ell}. \tag{17}$$

Using the Jacobi–Anger identity we expand the nonlinear term in Supplementary Eq. (16) up to third order

$$\begin{aligned}
&\sin\left(\varphi_1(t)\sin\frac{\pi x}{\ell}+\varphi_3(t)\sin\frac{3\pi x}{\ell}\right)= \\
&2\sin\frac{\pi x}{\ell}\Big(J_1[\varphi_1(t)]J_0[\varphi_3(t)]+J_2[\varphi_1(t)]J_1[\varphi_3(t)]\Big)+ \\
&+2\sin\frac{3\pi x}{\ell}\Big(J_3[\varphi_1(t)]J_0[\varphi_3(t)]+J_0[\varphi_1(t)]J_1[\varphi_3(t)]\Big).
\end{aligned} \tag{18}$$

Here we limited the series only to the first and third modes, $\sin\left(\frac{\pi x}{\ell}\right)$ and $\sin\left(\frac{3\pi x}{\ell}\right)$, in which we are currently interested. We consider the case of strong pumping of the first mode and weak probing of the third mode. Therefore, Supplementary Eq. (16) can be simplified and splits into two separate equations for each mode

$$\frac{\hbar}{2e}\left(C_0+\left(\frac{\pi a}{\ell}\right)^2 C_\mathrm{J}\right)\ddot{\varphi}_1+2I_\mathrm{c}\left(\frac{\pi a}{\ell}\right)^2 J_1[\varphi_1]=2\frac{a}{\ell}I_1\cos(\omega_a t), \tag{19}$$

$$\frac{\hbar}{2e}\left(C_0+\left(\frac{3\pi a}{\ell}\right)^2 C_\mathrm{J}\right)\ddot{\varphi}_3+I_\mathrm{c}\left(\frac{3\pi a}{\ell}\right)^2\left(1-\frac{\varphi_1^2}{4}\right)\varphi_3=-2\frac{a}{\ell}I_3\cos(\omega t). \tag{20}$$

Since the first mode drive is much stronger than the third mode drive, the equation of motion of the first mode (19) contains only self-Kerr nonlinearity and looks like Supplementary Eq. (10). On the contrary, the equation of motion of the third mode (20) is linear in φ_3 and contains only cross-Kerr nonlinearity. In analogy to the self-Kerr coefficient derivation, we look for the solution of Supplementary Eq. (20) at the same frequency of the drive ω

$$\varphi_3=\varphi_b\cos(\omega t+\delta), \tag{21}$$

where φ_a is the amplitude of the third mode response for each JJ and δ is a phase delay due to losses in the system. Solving Supplementary Eq. (20) with ansatz (21) we obtain

$$\varphi_b=\frac{\frac{4e}{\hbar\left(C_0+\left(\frac{3\pi a}{\ell}\right)^2 C_\mathrm{J}\right)}\frac{a}{\ell}I_3}{\sqrt{\left(\omega^2-\omega_3^2\left(1-\frac{\varphi_a^2}{8}\right)\right)^2+\gamma^2\omega^2}}. \tag{22}$$

Again, we derive the resonance frequency by maximizing Supplementary Eq. (22)

$$\omega=\omega_3\sqrt{1-\frac{\varphi_a^2}{8}}\simeq\ \omega_3\left(1-\frac{\varphi_a^2}{16}\right), \tag{23}$$

which gives the cross-Kerr coefficient between the first and third modes

$$K_{13}=\frac{1}{4}\pi e a\frac{\omega_1\omega_3}{j_c V_\mathrm{grAl}}. \tag{24}$$

Performing the same procedure for first and all the other modes, we obtain the cross-Kerr coefficients

$$K_{1n}=\frac{1}{4}\pi e a\frac{\omega_1\omega_n}{j_c V_\mathrm{grAl}}. \tag{25}$$

Circuit quantization

The total Q_{total} and coupling quality factor Q_c can be extracted from the measurement, allowing the average number of photons in the resonator to be calculated. In the case of one port waveguide for the first resonance mode it can be written as

$$\bar{N} = P_{in} \frac{4Q_{total}^2}{\hbar\omega_1^2 Q_\mathrm{c}}, \tag{26}$$

where P_{in} is the input power at the cavity port. The average number of photons relates to the amplitude of the current circulating in the resonator as

$$I_{res}^2 = 2\pi \frac{a}{\ell} \hbar\omega_1 \bar{N}/L_J, \tag{27}$$

where $L_J = \frac{\hbar}{2eI_\mathrm{c}}$ is the Josephson inductance of one junction[1]. At resonance, φ_a reaches its maximal value, which is

$$\varphi_a = \frac{2\pi}{\Phi_0} I_{res} L_J, \tag{28}$$

where $\Phi_0 = h/2e$ is the (superconducting) magnetic flux quantum. The self-Kerr nonlinearity is proportional to the second order of the phase response

$$\varphi_a^2 = 4\pi e a \frac{\omega_1}{j_c V_\mathrm{grAl}} \bar{N} \tag{29}$$

Substituting Supplementary Eq. (29) in Supplementary Eq. (14) we obtain

$$\omega = \omega_1 - \frac{3}{16} \frac{\pi e \omega_1^2}{I_\mathrm{c}} \frac{a}{\ell} \bar{N} = \omega_1 - K_{11} \bar{N} \tag{30}$$

Details on samples

We measure the fundamental frequency of all resonators directly with the VNA. Due to its symmetry, the second mode is decoupled from the waveguide mode. For the two longest resonators made of grAl#1 film (4000 µΩ cm), the third mode, which is outside the frequency range of our VNA, can be excited by a second tone, generated by an RF generator, and detected via its cross-Kerr interaction with the first mode. Knowing the first and third resonance frequencies we use Eq. (3) of the main text to derive the plasma frequency

$$\omega_p = 2\omega_1\omega_3\sqrt{\frac{2}{9\omega_1^2 - \omega_3^2}}, \tag{31}$$

which for $f_1 = 6.287 \pm 0.001$ GHz and $f_3 = 18.255 \pm 0.001$ GHz gives $\omega_\mathrm{p} = 68 \pm 0.1$ GHz.

Figure 3a and b depict the measured S_{11} response in the single photon regime for the stripline resonator of 0.6 mm length and 0.04 mm width made of grAl#1 film. A circle fit routine[2] is used to extract the internal and coupling quality factors $Q_\mathrm{i} = 10^5$ and $Q_\mathrm{c} = 10^4$ respectively[3], and the resonance frequency $f_1 \approx 6.3$ GHz.

Supplementary Table 1. Details on the samples from Fig. 4. The resonant frequencies and the quality factors are extracted from measurements in the low frequency setup, in a strongly shielded enviroment. For sample grAl#3, listed on the last line, only measurements using the high frequency setup were performed, which does not allow an accurate measurement of the intrinsic (unloaded) eigen frequencies and quality factors.

ρ, µΩ cm	ℓ, µm	b, µm	V_{grAl}, µm^3	f_1, GHz	f_3, GHz	K_{11}^{exp}, Hz	Q_c	Q_i	T_c, K
2000	2.7	0.05	0.003	4.7031	-	29×10^3	6×10^3	3.3×10^4	1.99
2000	2.7	0.2	0.01	4.9555	-	7.4×10^3	5×10^3	1.1×10^4	1.99
4000	400	5.4	43.2	6.995	-	135	60×10^3	1.5×10^5	-
4000	600	10	120	6.287	18.255	21	10×10^3	1.5×10^5	-
2800	600	8.9	107	7.6139	-	18.8	20×10^3	3.0×10^4	-
2800	600	7.3	88	7.231	-	6	12×10^3	8.0×10^4	-
4000	1000	40	800	6.024	17.645	5	4×10^3	1.5×10^5	-
2800	1000	31.2	624	8.635	-	0.9	4×10^3	2.8×10^4	-
1600	1200	12	288	3.16	-	1.1	24×10^4	1×10^4	2.03
900	1200	12	288	3.51	-	0.2	20×10^4	1×10^4	2.13
40	2500	2	100	5.18	-	65×10^{-3}	12×10^4	0.2×10^4	1.9
80	2500	2	100	4.12	-	26×10^{-3}	31×10^4	3×10^4	2.04
220	2500	2	100	2.58	-	22×10^{-3}	31×10^4	3×10^4	2.17
160	2500	2	100	2.57	-	18×10^{-3}	30×10^4	3×10^4	-
3000	1200	12	432	4.0	-	-	-	-	1.91

An overview of samples holders and sample geometries is provided in Supplementary Fig. 2 (in this appendix).

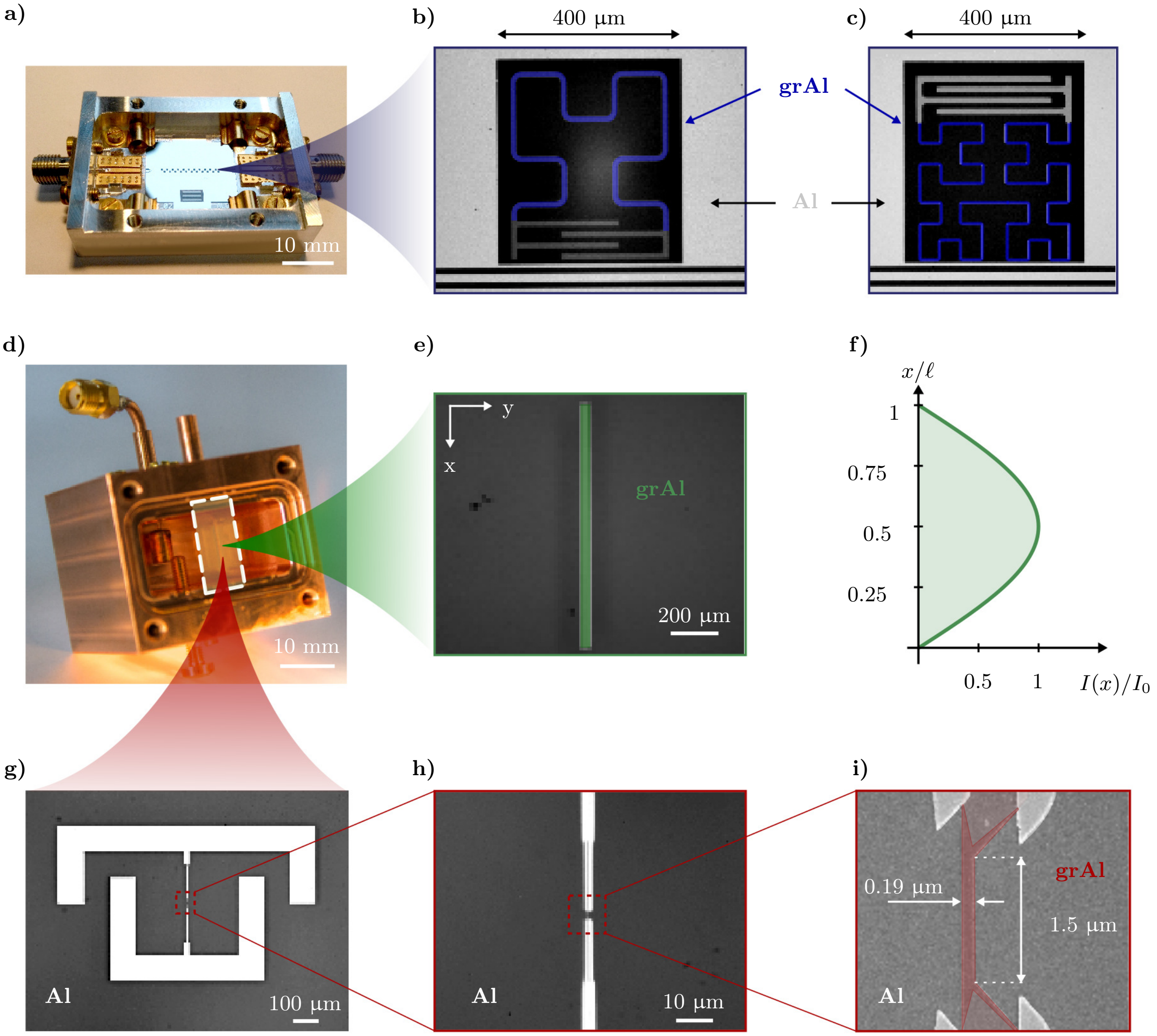

Supplementary Figure 2. **(a)** 2D sample holder for reflection and transmission measurements of microwave resonators which are coupled to an on-chip feedline in a notch-type geometry. **(b)**, **(c)** Lumped element resonators fabricated from granular aluminum (grAl) in a standard KID geometry with inductors in a Hilbert 2 and Hilbert 3 meander shape, respectively, and interdigitated shunt capacitors. The resonators are either coupled capacitively or inductively to the aluminum CPW transmission line. For both samples, the grAl volume contributing to the total kinetic inductance of the fundamental mode and, thus to the self-Kerr coefficient K_{11}, is highlighted in blue. Although the inductor in the Hilbert 3 shape is much longer, the smaller wire width results in an overall smaller total volume. **(d)** 3D sample holder for reflection measurements. **(e)**, **(f)** Rectangular shaped distributed microstrip-stripline resonator with open boundary conditions fabricated from grAl. The whole resonator volume contributes to the kinetic inductance (green shaded area), and the shape of current profile is accounted by $\mathcal{C}$ in Eq. (4). **(g)** Aluminum-shunted lumped element grAl resonator formed by two large aluminum islands connected via a thin bridge. **(h)** The kinetic inductance of pure aluminum and the geometric inductance of the bridge are neglectable, only a small un-shunted volume of highly resistive grAl in the center of the bridge is contributing to the total inductance of the resonator. **(i)** The zoom-in into the bridge center shows the un-shunted grAl film, shaded in light red, and the total grAl volume contributing to the kinetic inductance according to the expected current flow, shaded in dark red.

Martin-Puplett Interferometer (MPI)

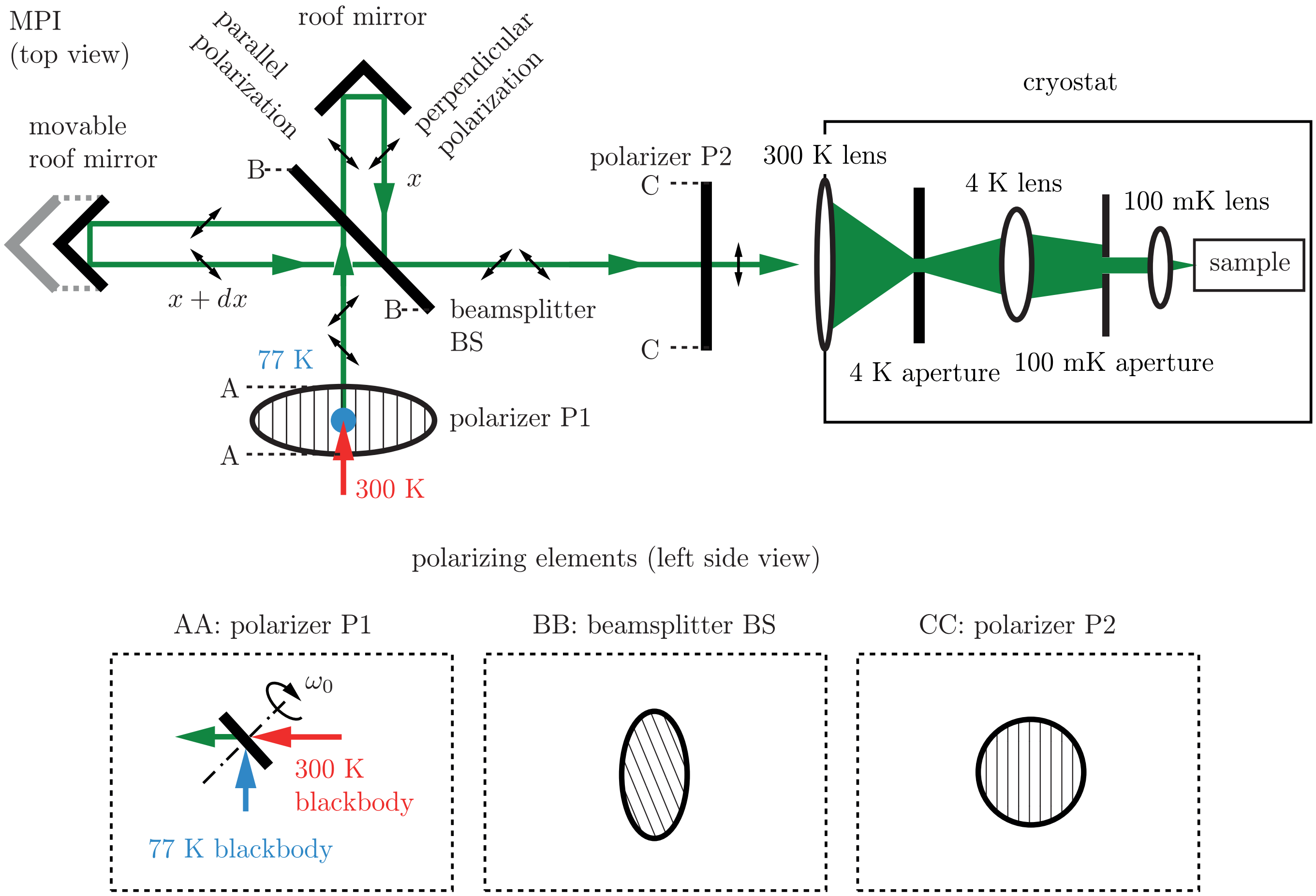

Supplementary Figure 3. **Optical diagram of the Martin-Puplett interferometer.** The source radiation is two combined beams from two blackbodies at 77 K (liquid Nitrogen) and 300 K. The rotating polarizer P1 combines and polarizes the beam, which is then divided by two partial beams by the beam splitter BS. The wire grids of the beam splitter BS are oriented at 45° to the normal of the drawing so that the polarization component perpendicular to the grid is transmitted and the component parallel to the grid is reflected. Two roof mirrors (one of each is movable) bring two components back to BS with a 90° rotation of the polarization. If the two roof mirrors are equally spaced from BS, the input and output beams of the BS are polarized identically. After polarizer P2 only one orthogonal polarization is transmitted to the cryostat. The cryostat optical system consists of a lens at room temperature, and two aperture and lens pairs, at 4 K, and at 100 mK, in front of the sample.

We use the Martin-Puplett interferometer (MPI) as a broad-band illumination source, with a resolution up to 1 GHz. A schematic drawing of the MPI is shown in Supplementary Fig. 3. Three wire grids are used as polarizer P1, beam splitter BS and polarizer P2. If the polarization of an incident wave is parallel to the wires, the wire grid behaves like the surface of a metal and the wave is reflected, whereas it would be a perfectly transparent element for a wave that is polarized orthogonally to the wires. The source radiation, emitted from two black bodies, at room temperature (red) and liquid nitrogen temperature (blue), is combined on the polarizer P1, which is a wire grid inclined by 45° with respect to the plane of the drawing. The polarizer P1 is rotating with frequency ω_0 about its axis, which creates the output beam containing two orthogonal polarizations that are swept over all possible orientations by a full rotation of P1. In the following discussion we'll consider P1 at a fixed moment in time. The beam is divided into two partial beams by the beam splitter BS, which is a fixed wire grid at a 45° angle to the incoming beam. The component of the incident beam with polarization parallel to the wires of BS is transmitted towards the fixed roof mirror, and the orthogonal component is reflected towards the movable roof mirror. The roof mirrors reflect the incident beams and flip their polarizations by 90°. The movable roof mirror can be displaced by an amount dx. The two beams recombine at BS with an accumulated path difference $\Delta = 2dx$, resulting in a phase difference $2\pi\Delta/\lambda$ which gives rise to interference. After the reflection both polarizations are flipped with respect to their first encounter

with BS, therefore the transmission/reflection routine is inverted. The polarizer P2 provides a reference for the ω_0 modulation of the polarization produced by P1. The single polarization output beam enters the cryostat through the room temperature lens. Here, the incoming beam is collected and sent to the cold optics, consisting on two additional focusing lenses, an in-focus aperture to reduce the intensity, and an out-of-focus aperture to crop the image. The intensity of the on-sample radiation for a single wavelength is[4]

$$I(\Delta) \propto I_0(\lambda)(1+\cos(2\pi\Delta/\lambda)), \tag{32}$$

where I_0 is defined among others by the diameter of the apertures and position of the sample with respect to the optical exes of cryostat optical system. $I(\Delta)$ is an even, periodic function of the roof mirror displacement, with the first maximum appearing at the origin, which is result of the alternatingly constructive and destructive interference.

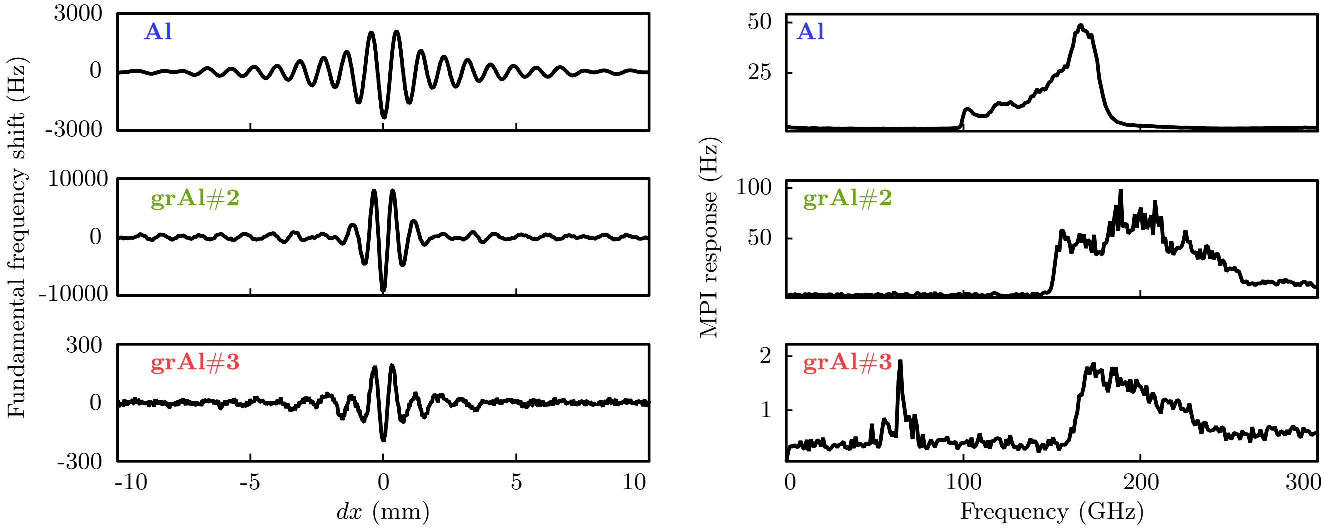

Supplementary Figure 4. Interferograms (left) and relative spectra (right, also present in the main text) of Hilbert-shaped resonators made of Al, grAl#2, and grAl#3. The black line shows the averaging over for nominally identical resonators at each sample.

The interferogram is the modulated term of Supplementary Eq. (32) integrated over all wavelengths. The parity of the integrand allows us to recast the cosine modulation as an exponential having the same argument, thus showing that the interferogram and the spectrum are Fourier transform (FT) pairs. The interferogram has a global maximum at the origin since different wavelengths will interfere in a fully constructive fashion only in the case of zero path difference. Furthermore, the interferogram is a stronger signal than the monochromatic intensity, since it encodes contributions from all wavelengths. Interferograms and their corresponding spectra (MPI responce versus illumination frequency) are shown in Supplementary Fig. 4.

The interferogram is generated by recording the on-sample irradiation at roof mirror steps $T_x \sim 10$ µm. This is equivalent to multiplying the a priori continous signal with a comb of Dirac deltas with spacing T_x. The FT of this product is a convolution of the spectrum with a $1/T_x$ Dirac comb in impulse space, i.e. an array composed by images of the spectrum spaced $1/T_x$ apart. The images are symmetrical and bounded by some $\pm f_{\max}$. The highest frequency that can be attained before image overlapping and subsequent aliasing is given by step T_x, for $T_x \approx 50$ µm the highest frequency $f_{max} = c/2T_x \approx 3000$ GHz. For all measurements presented in Supplementary Fig. 4 lowpass filters are used, for Al samples the cutoff frequency is 180 GHz, for grAl#2 and grAl#3 the cutoff frequency is 300 GHz

Critical current

The geometry and the switching current measurements for a DC-SQUID made with granular aluminum are presented in Fig 5. The measurements over 4 samples with resestivities 250, 1520, 3200 and 5550 μΩ·cm are summerized in Supplementary Fig. 6.

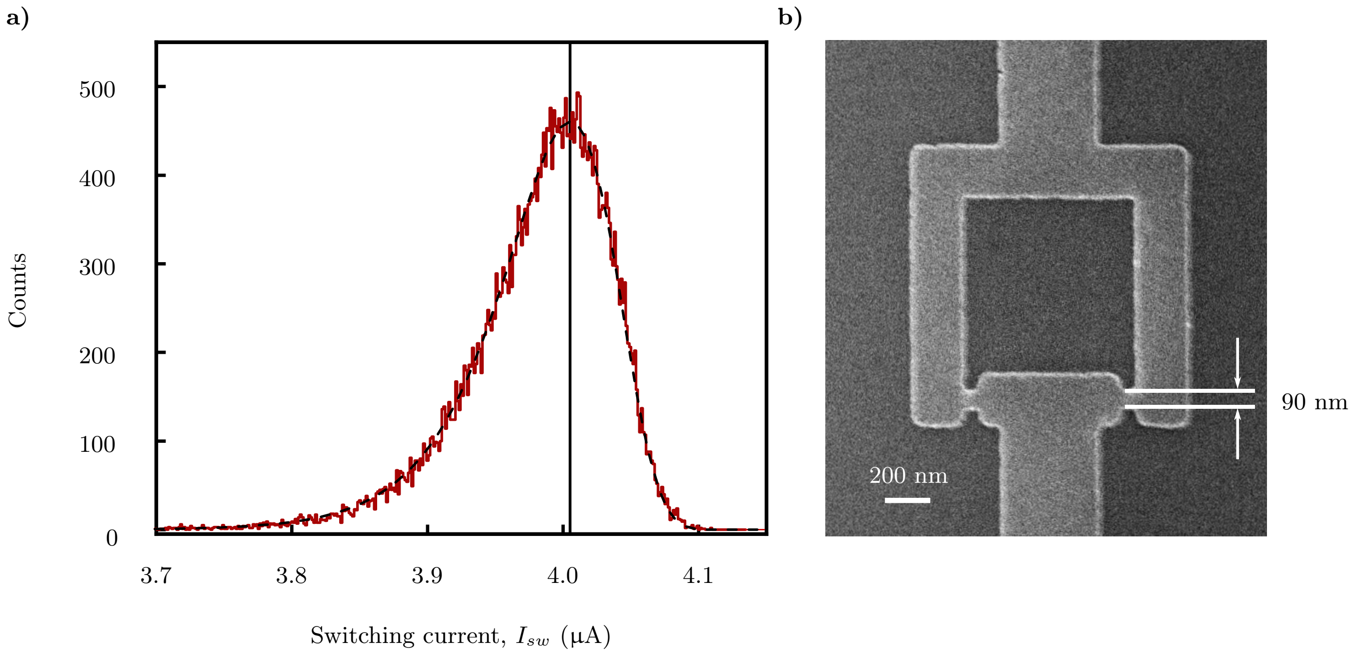

Supplementary Figure 5. **Switching current distribution of a grAl DC-SQUID. (a)** Switching current histogram at effective zero field for the SQUID shown in (b). The red curve shows the measured data. For each measurement, the bias current through the SQUID is increased at a constant rate until a finite voltage drop across the SQUID is detected. The applied current at this switching point defines I_{sw}. From a fit to the data (black dashed line) according to the expected switching probability distribution a mean value of 4.01 μA is obtained (for details on the fitting curve see[5,6]). **(b)** SEM image of the respective SQUID. The sample design was patterned on a Si/SiO_2 wafer by e-beam lithography followed by the evaporation of a 20 nm thick grAl thin film with a sheet resistance of 1600 μΩ·cm. The two SQUID junctions have a combined cross section of $2 \times 90\,\mathrm{nm} \times 20\,\mathrm{nm}$, resulting in a critical current density $j_{\mathrm{c}} \approx 1.1\,\mathrm{mA}\ \mu\mathrm{m}^{-2}$, quoted in the main text.

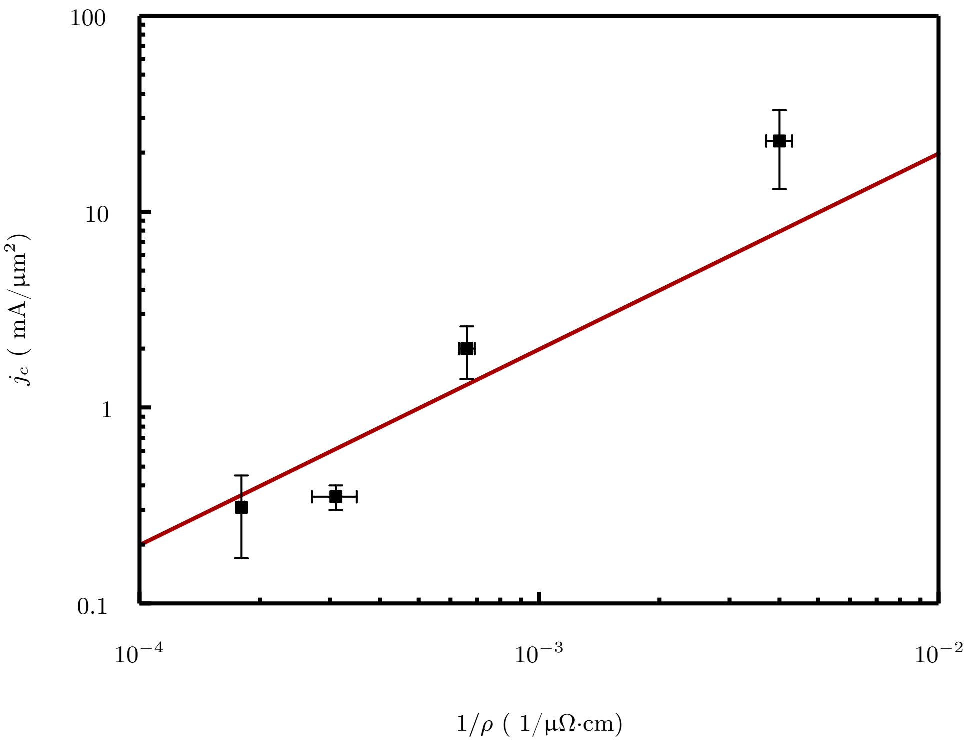

Supplementary Figure 6. **Measured critical current density j_c as a function of the inverse grAl normal state resistivity ρ.** Switching currents of several SQUIDs were measured for each resestivity point at a temperature of 20 mK. The geometries of the constrictions were extracted from SEM images, see Supplementary Fig. 5, to calculate the current density. The error bars indicate the standard deviation of the measured resistivites and switching current densities, respectively, over each ensemble of samples. A red line shows the linear scaling used in Supplementary Fig. 4 of the main text.

[1] M. H. Devoret, A. Wallraff, and J. M. Martinis, arXiv:cond-mat/0411174 (2004).
[2] S. Probst, F. B. Song, P. A. Bushev, A. V. Ustinov, and M. Weides, Review of Scientific Instruments **86**, 024706 (2015), http://dx.doi.org/10.1063/1.4907935.
[3] L. Grünhaupt, N. Maleeva, S. T. Skacel, F. Levy-Bertrand, A. V. Ustinov, H. Rotzinger, A. Monfardini, and I. M. Pop, arXiv:1802.01858 (2018).
[4] D. Martin and E. Puplett, Infrared Physics **10**, 105 (1970).
[5] D. Bluhm, *A deconvolution method for switching current histograms as a fast diagnosis tool*, Ph.D. thesis, The Graduate School, Stony Brook University: Stony Brook, NY. (2008).
[6] J. Kurkijärvi, Phys. Rev. B **6**, 832 (1972).
